# COVID-19 and rheumatic diseases: A mini-review

**DOI:** 10.3389/fmed.2022.997876

**Published:** 2022-09-26

**Authors:** Livia Roseti, Brunella Grigolo

**Affiliations:** SSD Laboratorio RAMSES, IRCCS Istituto Ortopedico Rizzoli, Bologna, Italy

**Keywords:** COVID-19, reactive arthritis, musculoskeletal pain, inflammation, infection, rheumatic diseases

## Abstract

Joint pain and arthralgia can be manifestations of COVID-19, and studies evaluating long COVID symptoms identified the persistence of these disorders. Moreover, some case reports highlighted the development of new inflammatory arthritis in patients with COVID-19, suggesting a possible relation. Viral infections and rheumatic diseases share a documented relationship; they have been associated with genetic and environmental risk factors responsible for some of them. There is crosstalk between viruses and the immune system during the development of several rheumatic diseases. Moreover, infections may participate in the pathogenesis of autoimmune rheumatic diseases and contribute to patient mortality. Therefore, it is crucial to provide a clearer insight into the interaction between viral infections and rheumatic diseases. Here, we provide a mini-review of the current literature with the aim of shedding light on the relationship between COVID-19 and rheumatic or musculoskeletal diseases, which is still unclear. Specifically, we examined several aspects: risk for the rheumatic population of acquiring the virus or developing severe symptoms, similarities of COVID-19 and arthritis, the possible rheumatic consequence of COVID-19, of rheumatic drugs and vaccines, and COVID-19 prevention in rheumatic patients through vaccination.

## Introduction

A novel coronavirus known as Severe Acute Respiratory Coronavirus-2 (SARS-CoV-2), which unexpectedly arose in December 2019, is still troubling the entire human population and has affected healthcare systems and the global socioeconomic balance. The resulting pathology, coronavirus disease-2019 (COVID-19), was quickly designated as a global pandemic by the World Health Organization and, to date, has resulted in millions of infections and deaths (1). Since then, numerous new strains of SARS-CoV-2 have emerged, showing increased transmissibility and resistance to therapies. Vaccine development in 2021 was a watershed in COVID-19 management, at least in the countries where vaccinations have been administered on a large scale. With still no effective treatment for COVID-19, vaccination seems to be the most effective method of prevention (2). While all coronaviruses distress the respiratory tract, SARS-CoV-2 can also affect the heart, gastrointestinal system, liver, kidneys, and central nervous system, eventually leading to multi-organ failure (3, 4). There has been a significant advancement in gaining information about SARS-CoV-2. However, there are still many issues to explore, especially to understand the consequences of the infection on individuals, both post-COVID (short term) and long COVID (long term), specifically as local and systemic pathophysiological outcomes (1). This pandemic has also led to more concern about people with an immune or inflammatory rheumatic disease. It seems essential to understand if these patients are more prone to SARS-CoV-2 than the general population and if their immunological alterations [as a direct result of rheumatic diseases (RD) or an indirect effect of treatment] have the potential to contribute to the development of severe COVID-19 (5, 6). At the beginning of COVID-19, several rheumatology organizations recommended following local public health guidance to reduce contagion risk: social distancing, hand washing, wearing masks, and isolation. Regarding drug management for these patients, the use of many conventional synthetic disease-modifying anti-rheumatic drugs (csDMARDs) and biological DMARDs (bDMARDs) does not necessarily mean a higher risk of poor outcomes with COVID-19 (7–9). Patients should continue their therapy in the absence of ongoing or suspected SARS-CoV-2 infection (10).

Viral infections and RD share many features. A few viruses have been associated with genetic and environmental risk factors for some RD. There is crosstalk between viruses and the immune system during developing several RD. Moreover, infections may participate in the pathogenesis of autoimmune RD and contribute to patient mortality. Therefore, it is crucial to provide a clearer insight into the interaction between viral infections and rheumatic disorders (11).

Here, we provide a mini-review of the current literature to shed light on the relationship between COVID-19 and rheumatic or musculoskeletal diseases, which is still unclear. Specifically, after a brief presentation of COVID-19 and RD, we examined several aspects: risk for the rheumatic population of acquiring the virus or developing severe symptoms, similarities of COVID-19 and arthritis, the possible rheumatic consequence of COVID-19, of rheumatic drugs and vaccines, and COVID-19 prevention in rheumatic patients through vaccination.

### Search strategy and manuscript selection

We performed a comprehensive search according to the review strategies recommended by Gasparyan et al. (12). We conducted the search through LitCovid/Pub-Med, Scopus, and Web of Science databases, using the keywords “COVID-19,” “COVID infection,” “post COVID arthritis,” “post COVID musculoskeletal pain,” “reactive arthritis,” “rheumatoid arthritis,” “osteoarthritis,” “spondyloarthritis,” “autoimmune disorders,” “rheumatic disorders,” “rheumatic diseases,” “COVID-19 vaccine,” and “COVID-19 vaccination.” We reviewed 120 articles and included 87. Non-English sources, conference abstracts, and non-peer-reviewed sources were not included.

## COVID-19

The COVID-19 pandemic is a world public health threat caused by SARS-CoV-2, with millions of people at risk in many countries. Transmission may occur through infectious respiratory droplets or direct or indirect contact. Infection involves mainly the respiratory tract or becomes systemic, with a spectrum of symptoms. Since the beginning of the pandemic, several virus variants have emerged. They differ in transmissibility, disease severity, and ability to escape humoral immunity (13).

**Figure 1:**
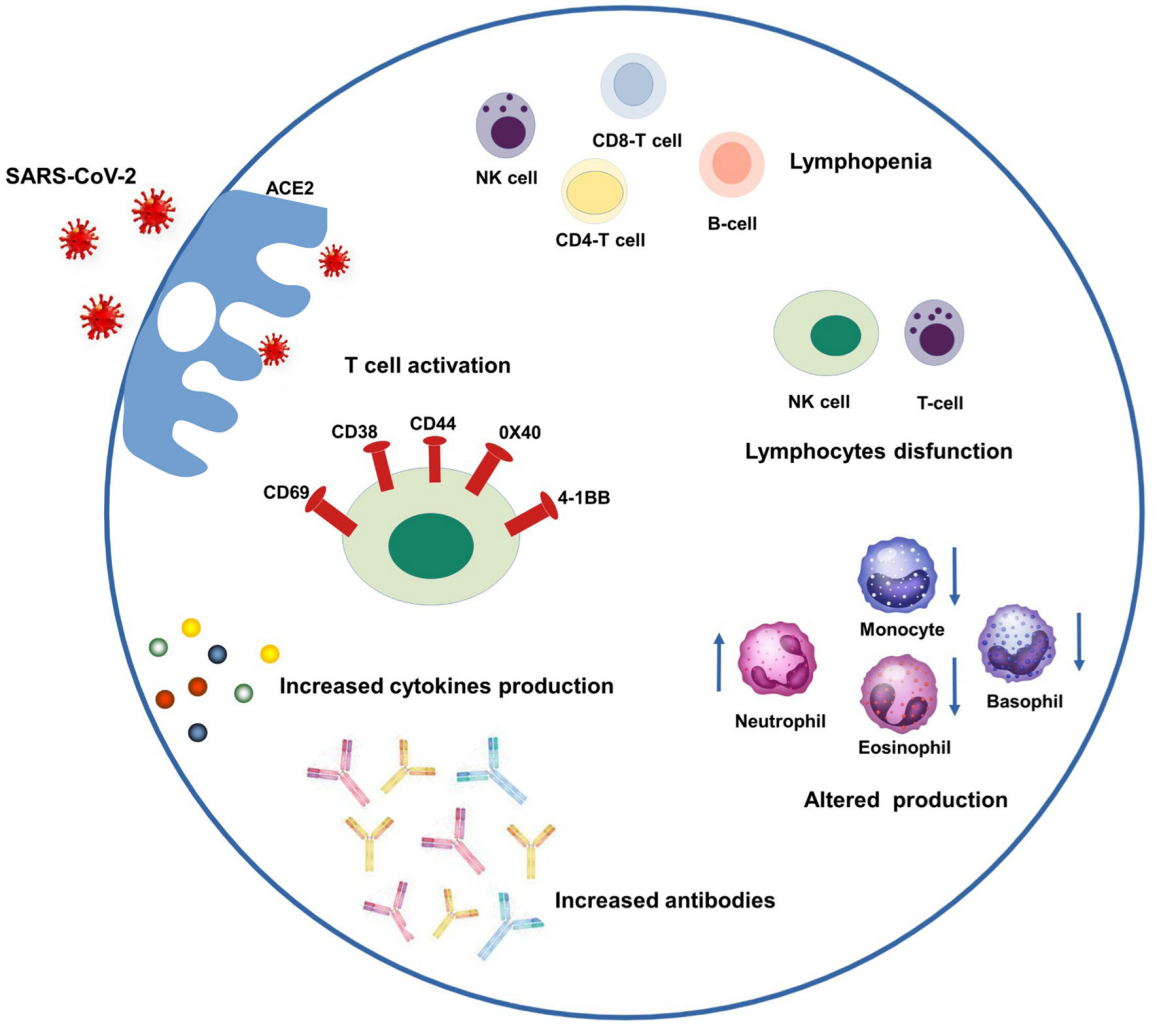
Schematic representation of COVID-19 immune patterns. In the left-upper part of the scheme, SARS-CoV-2 enters the host's cells through the ACE-2 specific receptor and triggering several patterns. Clockwise from the left they are: T cell activation through a high expression of CD69, CD38, and CD44, 0X40 and 4-1BB; lymphopenia including NK, CD4-T, CD8-T and B-cells; lymphocyte dysfunction including NK and T-cells; monocytes and granulocyte abnormalities (increased neutrophil levels, decreased eosinophil, basophil, and monocyte levels), increased amount of antibodies and cytokines.

SARS-CoV-2 binds to epithelial cells in the oral and nasal cavities and can migrate further down the respiratory tract into the conducting airways. It enters the host's cells through the angiotensin-2 converting enzyme (ACE-2) specific receptor. ACE-2 is a type I membrane glycoprotein expressed in the lungs, nose, heart, intestines, and kidneys. It converts angiotensin II into angiotensin-(1–7) and, if unregulated, an imbalance between the renin-angiotensin system and ACE2/angiotensin-(1–7)/MAS after infection may contribute to multiple organ injury (14, 15). The immune patterns of COVID-19 include lymphopenia, lymphocyte activation and dysfunction, granulocyte abnormalities, cytokine and monocyte production, and increased antibodies. Lymphopenia is a crucial feature of patients with COVID-19, especially in severe cases. There is a high expression of CD69, CD38, and CD44 on CD4+ and CD8+ T cells and virus-specific T cells from severe cases exhibit a central memory phenotype with high levels of Interferon gamma (IFN-γ), tumor necrosis factor-alpha (TNF-α), and Interleukin-2 (IL-2). However, lymphocytes show an exhaustion phenotype with programmed cell death protein-1 (PD1), T cell immunoglobulin domain and mucin domain-3 (TIM3), and killer cell lectin-like receptor subfamily C member 1 (NKG2A) upregulation. Neutrophil levels are high in severe patients, while eosinophil, basophil, and monocyte levels are low. Increased cytokine production, especially IL-1β, IL-6, and IL-10, is another critical characteristic of severe COVID-19. IgG levels are also increased, with a higher titer of total antibodies (Figure 1) (16–19). A spectrum of symptoms involving several organs can continue or develop after acute COVID-19 infection, leading to a condition known as long COVID (20, 21). Several drugs specific to COVID-19 or typically used for other diseases have been administered, and behavioral measures have been adopted. Authorized vaccines against COVID-19 allow proper pandemic management (13, 22).

## Rheumatic diseases

RD are a broad spectrum of disorders that can affect many tissues, with the predominant involvement of the musculoskeletal system. RD imply pain and disability in the population, with health, social and economic burdens. The pathogenesis is multifactorial: immune complexes, autoantibodies, and abnormal T-lymphocyte responses all play a part. Genetic predispositions, environmental agents, and sex hormones also contribute. Smoking, obesity, alcohol consumption, injury, and work activities are associated with risk for at least one rheumatic disease. Medical treatments enclose glucocorticoids, methotrexate, other disease-modifying agents, and non-pharmacologic strategies like surgery and physical and occupational therapy (22, 23). Joint inflammation is a significant trait of RD. Inflammation is a vital cellular process or an immune response to injury, tissue damage, or body infection, maintaining tissue homeostasis under traumatic or stressed conditions and regulating the host's defense mechanism against pathogens (12). There are many mediators of inflammation enclosing Cytokines [Interleukins (ILs) like IL-1, -6, and -8; tumor necrosis factor-alpha (TNF-α); chemokines], enzymes (cyclo-oxygenases COX-1, and -2; matrix metalloproteinases MMP-9; 5-lipooxygenase 5-LOX), inducible nitric oxide synthase (iNOS), transcription factors (Signal transducer and activator of transcription 3, STAT3), and nuclear factor kappa-B (NF-κB). Several viruses are associated with acute or chronic arthritis, like parvovirus, Chikungunya, Zika, Hepatitis B and C, Epstein-Barr, and Human Immunodeficiency Virus. Arthritis may result from the direct invasion of the joints or changes in the immune system. Among the different etiopathogenetic mechanisms, molecular mimicry has been the most emphasized (1, 24, 25).

The classification of RD is sometimes difficult due to the frequent lack of defined etiological evidence, the heterogeneity of symptoms, and the considerable clinical and pathological overlap between many rheumatic conditions. A simple classification is based on their nature and encloses autoimmune, autoinflammatory, and degenerative/metabolic disorders. Autoimmune RD impair the balance between avoidance of self-attack and recognition of pathogens, and the immune system is continuously active in the absence of infection. Autoinflammatory RD consist of unprovoked inflammation episodes without antigen-specific T cells or high-titer autoantibodies caused by dysregulation of innate immunity. Degenerative arthritis is synonymous with osteoarthritis, a chronic disorder that damages the joint. Metabolic disorder in bone may lead to osteoporosis and articular and periarticular deposits of calcium pyrophosphate, hydroxyapatite, and monosodium urate (26).

Among RD osteoarthritis (OA), rheumatoid arthritis (RA), and ankylosing spondylitis account for a large percentage of pain and disability worldwide and are the most common in clinical practice (23). The pathological traits of OA consist of articular cartilage degradation, subchondral bone thickening, osteophyte formation, synovial inflammation, ligament degeneration, and capsule hypertrophy. OA is a diffused chronic joint disease characterized by pain, deformity, instability, and reduced motion and function. Unlike focal defects, which usually involve a younger population who suffered an acute trauma and require localized treatment, OA lesions often affect elderly patients and the whole joint surface (24, 27). RA is a common chronic, systemic, autoimmune, inflammatory disease, primarily affecting the joints. Patients are generally seropositive for immunoglobulin G and citrullinated proteins; some individuals are seronegative. RA etiology is currently unknown. Risk factors are genetic and environmental. The processes involved in the disease are multiple and complex: T- and B-cells, macrophages, fibroblasts, Reactive Oxygen Species, and cytokines mediate inflammation (28, 29). Ankylosing spondylitis is a chronic inflammation of the spine (including sacroiliac joints), a high percentage of enthesopathy, a tendency of familial aggregation, and an association of human leukocyte antigen (HLA)-B27 (26). As with other viral infections, COVID-19 joint involvement may have the traits of reactive arthritis (ReA). ReA is a spondyloarthritis occurring mainly after genitourinary or enteric infection. It derives from a tissue infection further away from the joint rather than a direct articular infection. ReA is frequent in young male adults. It can be mono- or oligo-articular and involves large joints. ReA generally resolves shortly but can assume a chronic form (1, 25, 30).

## The relationship between COVID-19 and rheumatic diseases

### Risks for the rheumatic population of acquiring COVID-19 or developing severe symptoms

When the COVID-19 pandemic began, it was unknown if the rheumatic population would be more prone to acquiring the virus or developing severe symptoms than the general population. In a Spanish study, patients with RD were more susceptible to COVID-19. The cumulative incidence was five times higher than in the general population, and treatment with anti-TNF-alpha was associated with increased SARS-CoV-2 infection risk (31). However, in an Italian study, the seroprevalence and titer of SARS-CoV-2 antibodies in patients with inflammatory arthritis receiving DMARDs were comparable with the healthy population. Glucocorticoids and comorbidities were associated with a higher seroprevalence rate in these patients (32). Therefore, even if people with RD seem to have some increased risk of complications from COVID-19, this might be due to comorbidities (5, 25, 33). Thus, the risk factors for COVID-19 infection or developing severe illness in the general population may also apply to patients with rheumatic disease.

### Similarities of COVID-19 and arthritis

Although there is evidence of musculoskeletal manifestations involving immune-inflammation-dependent mechanisms and cases of arthralgia and/or myalgia within SARS-CoV-2 (34), common features and crosstalk between COVID-19 and immune-based RD are still debated. Some similarities have been recently reported between COVID-19 and OA patients like hypocalcemia, vitamin D deficiency, endothelial and adipose tissue dysfunction, neuronal sensitization, and joint and muscle pain (35, 36).

The recent data highlight that COVID-19 and RA share similar immuno-inflammatory pathways, pro-inflammatory mediators, and the progression into a “cytokine storm” (17, 37). IL-6 plays a critical pathophysiological role in both pathologies. In RA, it is responsible for joint damage. In COVID-19, it is correlated with increased severity of lung disease and patient mortality (38). Even IFNs, IL-1β-, IL-6, and TNF-α-related pathways are involved in similar ways, (39) presenting aberrant ACE/ACE2 activity and analogous macrophage clusters (40). Moreover, SARS-CoV-2 activates CD14 + monocytes and Programmed death-ligand 1 positive (PD-L1+) neutrophils through the osteopontin-mediated inhibition of IL-10. Additionally, as already mentioned, various autoantibodies have been reported in COVID-19 (21). Due to certain similarities between COVID-19 and RA, some treatments used to treat COVID-19 patients have been borrowed from rheumatologists: hydroxychloroquine, glucocorticoids, IL-6, IL-1 inhibitors, and anti-TNF and Janus Kinase (JAK) inhibitors. It was rapidly evident that hydroxychloroquine was ineffective. As for the other therapeutical agents, the implications for COVID-19 patients are unclear. Glucocorticoids have a dose-dependent effect on serious infection risk. Anti-TNF and JAK inhibitors are associated with an increased infection risk as well (5, 6, 41, 42).

### Rheumatic diseases after COVID-19 infection: Incidence and manifestation

Like many other viral infections, SARS-CoV-2 can potentially lead to rheumatological and autoimmune manifestations such as arthralgia, myalgia, proximal weakness, disabling fatigue, musculoskeletal pain, cognitive difficulties, and mood. The immune consequences of SARS-CoV-2 infection and the environment should explain the rheumatic musculoskeletal manifestations of COVID-19 (43).

Many patients with rheumatic and musculoskeletal diseases continue to have persistent and/or increased symptoms, especially fatigue, and dyspnea. It is important understanding the potential pathways and manifestations leading to rheumatic illnesses that could be triggered due to COVID-19. In a prospective study, Di Iorio et al. (44) describe a sequela of worse events after COVID-19 in patients with systemic autoimmune RD, possibly related to underlying condition leading to altered immunity, propensity for hyperinflammation, immunosuppression, organ damage, and comorbidities.

Although there are only a few case reports or series, it seems that COVID-19 patients may develop new ReA (1, 24, 28, 45–47). A definite clinical picture has not emerged, and some authors suggest the term “COVID-19 associated arthritis” (48). The median age is 50, higher than the typical age for ReA, and incidence is high in the male population, as it is for ReA (1, 24, 28). Part of the described patients with post-COVID-19 ReA present a rheumatoid-like phenotype (1, 24, 28). However, an elbow monoarthritis accompanied by psoriasis-like skin plaques (49), a spondyloarthritis-like presentation with axial involvement (50), and four cases of crystalline arthritis (51) have also been described. Moreover, multi-system inflammatory syndrome in children (MIS-C) was reported (52). Knee, wrists, ankles, and proximal interphalangeal joints are the most affected, consistent with ReA (1, 24, 28). Patients are mainly seronegative, but anti-citrullinated antibodies and antinuclear antibodies with a speckled pattern have been reported (20, 24). Involvement is generally mono- oligo- or poly-articular, without detectable erosive bone changes (1, 24, 28). Polyarticular ReA post-COVID-19 is compatible with viral-associated arthritis, which occurs with a polyarticular pattern like rheumatoid arthritis (1). Additionally, a few studies reported other post-COVID-19 rheumatic manifestations like carpal or tarsal tunnel syndromes (19, 53).

### Rheumatic musculoskeletal manifestations due to antiviral drugs

To date, no anti-viral or anti-inflammatory treatments are effective for SARS-CoV-2 infection since several studies have shown contradictory results. Moreover, due to the high number of infected people and the high SARS-CoV-2 infectivity and/or lethality, most therapeutic decisions have been made based on experience instead of evidence.

There is some evidence that some anti-viral drugs can result as well in several rheumatic musculoskeletal adverse effects. Chloroquine and hydroxychloroquine can cause myopathy and neuromyopathy, although current results do not seem to support their use in COVID-19 treatment anymore (54). Lopinavir, Ritonavir, and Ribavirin may cause arthralgia and back pain (55, 56). Musculoskeletal pain and myalgia have occurred in patients on IFN therapy (57). Active pharmacovigilance is essential to monitor a rise in these adverse reactions due to the use of these and other new drugs (43).

### COVID-19 prevention in rheumatic patients through vaccination

Rheumatic patients, particularly with immune-mediated inflammatory disease ones (IMID), are a priority target group for the COVID-19 vaccine campaign (58). In these patients vaccination induces an immunological response that is higlighted below.

#### Cellular and humoral response

There is little evidence on the COVID-19 vaccine-induced immune response, both cellular and humoral, and on the impact of variants. Recently, a study demonstrated that, after mRNA vaccination, IMD patients have a similar cellular immune response compared with immunocompetent subjects. However, stratifying patients based on the therapy regimen revealed a decreased T cell-specific response in patients taking cytotoxic T lymphocyte antigen 4 (CTLA-4)-IgG or TNF-α inhibitors. Regarding the T cell-specific response, the observed decrease is likely due to the well-known impact of CTLA-4-IgG on the downregulation of the antigen presentation, thus preventing T cell activation. In contrast, TNF-α is a crucial factor in all acute inflammatory reactions; therefore, its inhibition may have broader effects on T cell responses. Regarding the antibody response, no significant differences were observed for the anti-RBD IgG response, both quantitatively and qualitatively, between patients with IMID and immunocompetent subjects. We only found a reduced number of responders in patients under CTLA-4-IgG, although immunosuppressive therapies may impact the antibody response to anti-SARS-CoV-2 vaccines, as recently shown (58–62).

#### Outbreaks of rheumatic diseases after vaccination

Current evidence supports a non-significant flare-up in Rheumatic patients after COVID-19 vaccination. However, the risk of rheumatic disease flare or worsening does exist, especially in patients with high disease activity. COVID-19 vaccine may induce autoimmunity by similar SARS-CoV-2 infection mechanisms such as molecular mimicry, epitope spreading, bystander activation, and polyclonal activation (63).

Some studies have reported the flare rate of RD after COVID-19 vaccination, ranging from 0.4 to 20%, with a high level of heterogeneity due to vaccine types (Table 1) (64–68), number of administrations, and RD types. Most of the flares after COVID-19 vaccination were mild or moderate and needed no treatment escalation. The are predominantly presented as joint pain, stiffness, and swelling. Fatigue and myalgia were also commonly seen. Most flares happened quickly after the COVID-19 vaccination and were presented persistently within the first week. Most patients resolved shortly after the onset (generally within seven days), and only a small part needs treatment adjustment. The proportion of patients requiring hospitalization was meager (69–71). Some studies analyzed disease activity before and after vaccination and found no significant changes in the overall disease activity.

**Table 1:** Post-vaccination inflammatory arthritis.

**Vaccine**	**Rheumatic events**	**Reference**
CoronaVac	Left knee monoarthritis	(64)
Sinovac	Arthritis in the right wrist, 2nd−4th metacarpophalangeal and 2nd−4th proximal IP joints	(65)
Sinovac	Arthritis in left hand all distal IP joints; hip; entire spine	(65)
Sinovac	Arthritis in the left elbow, bilateral knees and right ankle	(66)
Sinovac	Arthritis in both wrists, hand joints, and left ankle	(66)
SPUTNIK-V	Left elbow	(67)
SPUTNIK-V	Arthritis in both shoulders and both knees initially. Involved small joints of hand and feet after the second dose	(68)

## Discussion

This mini-review aims to elucidate the relationship between COVID-19 and rheumatic or musculoskeletal diseases by highlighting several aspects: risk for the rheumatic population of acquiring the virus or developing severe symptoms, similarities of COVID-19 and arthritis, the possible rheumatic consequence of COVID-19, of rheumatic drugs and vaccines, and COVID-19 prevention in rheumatic patients through vaccination.

It seems that the rheumatic population is not at serious risk of acquiring the virus or developing severe symptoms any more than what is considered normal. Rheumatic or musculoskeletal conditions comprise many diseases and syndromes, usually progressive and associated with pain and reduced mobility (5–7). In addition, many of these conditions are autoimmune or autoinflammatory and might imply a higher possibility of SARS-CoV-2 infection, even if the additional risk is probably small. Comorbidities, drugs such as corticosteroids, and increased disease activity are more likely responsible for COVID-19 complications (10).

Furthermore, there is evidence that COVID-19 and RA share some pro-inflammatory pathways (33). The recent literature presents a few cases describing COVID-19 patients developing new ReA, mostly with RA traits. Those cases indicated that arthritis manifests after an acute COVID-19 infection; COVID-19 and RA share common pathways; many autoantibodies also characterize COVID-19; many human peptides have similarities with SARS-CoV-2 (1, 29, 31). However, besides being few cases, it is unclear if there is an unmasking of previous subclinical disease or virus-induced arthritis. Furthermore, a causal correlation between SARS-CoV-2 and ReA is not evident, and it could only be a coincidence, considering the high incidence of COVID-19 infections and RA (1, 12).

Vaccination for COVID-19 deserves special attention in rheumatic patients. The safety and efficacy of COVID-19 vaccination in patients with RD have been the object of debate. The supposed risks were whether immune responses could enhance SARS-CoV-2 acquisition or make the condition worse when infection occurs after vaccination. Furthermore, inflammatory musculoskeletal symptoms may occasionally develop with COVID-19 vaccine administration. Nevertheless, since no studies have demonstrated a significant risk of vaccine-induced autoimmunity, patients with RD should undergo vaccination against COVID-19, like the general population (4, 59). However, some precautions may be needed, especially considering some case reports or series of vaccine-induced autoimmune diseases (59) and the pathogenetic mechanisms of RD. The often-administered immunosuppressive drugs compromise vaccination responses potentially, and, notably, for bDMARDS like rituximab or tofacitinib (especially in combination with methotrexate), it may be appropriate to discontinue the therapy before and after vaccination (59). Finally, the last-generation mRNA-based vaccines are relatively new, not widely used, and may cause robust IFN responses, often associated with inflammation and autoimmunity. Therefore, rheumatic patients need close monitoring after vaccination (60). Another debate was whether it was preferable to vaccinate rheumatic patients in suboptimal circumstances (e.g., active disease, receiving high-dose glucocorticoids, receiving cytotoxic therapy) or wait until more clinical stability (stable or low disease activity). The European Alliance of Associations for Rheumatology (EULAR) recommended that patients with autoimmune/autoinflammatory RD not be administered vaccines during the clinical or serologically active period (61). Similar indications should also apply to COVID-19 vaccines. However, under the assumption that the vaccine would confer at least some protection, the American College recommended vaccination irrespective of disease activity and severity, except for those with severe and life-threatening illnesses (e.g., a hospitalized patient receiving treatment in the Intensive Care Unit for any condition) (70).

Optimizing the care of people with rheumatic pathologies during the COVID-19 pandemic is crucial. With this mini-review, we have tried to offer a succinct, up-to-date analysis of the relationship between COVID-19 and RD, focusing on critical emerging aspects. As a result, we found that many concepts are still unclear, several definitive support data are still lacking, and there are some gaps in the research. We believe that extensive, multicenter, international studies are necessary to investigate the mechanisms underlying the virus's interaction with the immune system, particularly regarding the potential association between SARS-CoV-2 infection and systemic autoimmune diseases. The limit of this mini review is that it is narrative. A systematic review starting from a formulated question would make it possible to extract and analyze data based on evidence and obtain a more comprehensive report. The advantage of a narrative review is the ability to discuss data, highlight gaps in knowledge or research, and identify future developments. We intended to provide the readers with a critical point of view on the crucial topic of managing rheumatic patients in the time of COVID-19.

The COVID-19 pandemic has proposed a significant challenge in monitoring rheumatic patients for all the above reasons. Some critical concerns on the relationship between SARS-CoV-2 and RD have been addressed. However, despite the increasing number of publications, knowledge gaps and open questions still exist. Many studies are heterogeneous, insufficient and mixed, in terms of population, treatments and vaccines. This makes data extrapolation difficult and may undermine conclusions. Moreover, various studies report that rheumatic patient adhered to isolation measures more strictly (5, 6). This fact may impact the absolute risk of acquiring COVID-19 and should be considered when interpreting the data (5, 6, 8–11).

## Author contributions

Conceptualization and writing review and editing: BG and LR. Methodology and writing original draft preparation: LR. Supervision: BG. All authors have read and agreed to the published version of the manuscript.

## Conflict of interest

The authors declare that the research was conducted in the absence of any commercial or financial relationships that could be construed as a potential conflict of interest.

## Publisher's note

All claims expressed in this article are solely those of the authors and do not necessarily represent those of their affiliated organizations, or those of the publisher, the editors and the reviewers. Any product that may be evaluated in this article, or claim that may be made by its manufacturer, is not guaranteed or endorsed by the publisher.
